# Model reduction enables Turing instability analysis of large reaction–diffusion models

**DOI:** 10.1098/rsif.2017.0805

**Published:** 2018-03-14

**Authors:** Stephen Smith, Neil Dalchau

**Affiliations:** 1 Biological Computation group, Microsoft Research, Cambridge CB1 2FB, UK; 2 School of Biological Sciences, University of Edinburgh, Edinburgh EH9 3JR, UK

**Keywords:** Turing patterns, model reduction, synthetic gene circuits, reaction–diffusion

## Abstract

Synthesizing a genetic network which generates stable Turing patterns is one of the great challenges of synthetic biology, but a significant obstacle is the disconnect between the mathematical theory and the biological reality. Current mathematical understanding of patterning is typically restricted to systems of two or three chemical species, for which equations are tractable. However, when models seek to combine descriptions of intercellular signal diffusion and intracellular biochemistry, plausible genetic networks can consist of dozens of interacting species. In this paper, we suggest a method for reducing large biochemical systems that relies on removing the non-diffusible species, leaving only the diffusibles in the model. Such model reduction enables analysis to be conducted on a smaller number of differential equations. We provide conditions to guarantee that the full system forms patterns if the reduced system does, and vice versa. We confirm our technique with three examples: the Brusselator, an example proposed by Turing, and a biochemically plausible patterning system consisting of 17 species. These examples show that our method significantly simplifies the study of pattern formation in large systems where several species can be considered immobile.

## 1. Introduction

How cells coordinate with one another to form regular patterns of alternate differentiated states is a foundational question in developmental biology [1]. Establishing general rules that biochemistry can follow to enable pattern formation could impact on our ability to understand and cure developmental disorders [2], construct synthetic organs/organoids [3] or enable synthetic biology applications to use multicellular self-organization [4–6]. While there are several mechanisms that are known to enable multicellular self-organization of regular patterns, such as the *french flag* model [7], we focus here on *diffusion-driven instability* (DDI) first described by Alan Turing [8]. He proposed that two ‘morphogens’ (intercellular signalling molecules) could enable tissues to produce regular patterns, and introduced a framework based on the reaction–diffusion equations that can establish when a given chemical system has pattern-forming potential. Later, Gierer & Meinhardt proposed that self-organization requires a self-enhancing activator, which also upregulates an inhibitor, forming a negative feedback, and further that the activator must diffuse more slowly than the inhibitor [9]. While an activator–inhibitor system is the simplest pattern-forming network, requiring only two chemical species but with differential diffusion, the introduction of a third (non-diffusing) species has been found to enable pattern formation when the morphogens have equal diffusion rates [10,11].

Despite the theory of Turing patterns having existed since the 1950s, only much more recently has compelling evidence emerged that suggests that Turing patterns are responsible for pattern formation in natural biological systems, including digit patterning [12,13] and fish skin colouring [14]. In most cases, it has been challenging to relate known biology involving many interacting species to simple 2- and 3-species networks for which analysis of DDI is more straightforward [13,15]. As such, it remains the subject of debate as to whether the examples of biological pattern formation cited above actually depend on DDI, or might arise due to other reasons. To help understand the biochemical mechanisms that can result in biological pattern formation, several articles have proposed constructing synthetic biochemical networks that are engineered to specifically implement pattern-forming behaviours, some based on Turing instability [16–19] but also other mechanisms [20–24]. Libraries of biological parts/components have now been compiled that have been demonstrated to be functional in specific cellular systems that are frequently used in synthetic biology applications (e.g. *Escherichia coli* and *Saccharomyces cerevisiae*). Knowledge of the functioning of these components could then be used to demonstrate how manipulating kinetic parameters influence the conditions for DDI, and alter pattern wavelength, in predictable ways. Establishing a close relationship between theory and experiment would then provide further evidence that Turing's mechanism can drive biological pattern formation. However, examples of synthetic biological circuits that can produce Turing patterns have yet to emerge, further raising the question of whether the Turing mechanism alone is sufficient to robustly generate regular patterns in a biological system.

Analysis of DDI for two species is now well-established [25], but quickly becomes more complicated with the introduction of additional (often non-diffusing) species [10]. While more theory and automated mathematical tools are now emerging that facilitate the analysis of DDI in general *n*-dimensional systems [11,26], it still remains a challenge when the underlying system is nonlinear, as is typically the case in biological systems. Therefore, it is not uncommon to start with a more detailed mathematical description of a chemical system, then attempt to reduce it to a simpler form while retaining the majority of the behaviour of the detailed model [16]. However, little analysis has emerged that establishes whether the conditions of DDI are preserved during a model reduction, despite it being observed that model reduction can change the required diffusion ratio for pattern formation [10]. One paper has shown that reaction–diffusion systems with a particular simple form can be reduced without impacting on the dynamics (and consequently the pattern-forming capabilities), but the result is not generalizable beyond a small subset of systems [27].

Many techniques have been established that reduce the size of ordinary differential equation (ODE) models, offering a starting point for interpreting the impact of model reduction on Turing pattern formation. Each technique is based on maximizing the fidelity between detailed and reduced models with respect to a specific property (see [28,29] for reviews of model reduction techniques). Some methods guarantee that equilibrium solutions (and their stability properties) are retained through a reduction, while others attempt to minimize the deviation of the transient behaviour of a specified model variable or variables, in response to a stimulus. Furthermore, some methods preserve the model co-ordinates/variables, while others do not. In biochemical systems, timescale separation techniques are often used, of which the most common are the quasi-steady-state approximation (QSSA) and the quasi-equilibrium (QE) assumption [28]. Both involve removing species that are *fast*, substituting the concentration of these species for functions of the dynamic species that are derived from equilibrium relationships arising from the full system.

In this paper, we investigate the question of whether model reduction can be applied to a chemical reaction network (CRN) in a manner that preserves Turing pattern-forming behaviour. In §2, we prove that if a reduced model forms patterns, then so does the corresponding full system (and vice versa), given that some easily checkable conditions are fulfilled. In §3, we confirm our results on three separate CRNs, including the Brusselator, and a synthetic gene network with 17 species. These examples show that the method developed in this paper allows for quick and easy Turing pattern analysis of complex chemical systems with an arbitrarily large number of non-diffusible species.

## 2. Theory

### 2.1. Background description of Turing instability

The majority of theoretical work on Turing patterns builds upon the classical reaction–diffusion equations for a chemical system undergoing diffusion. In the absence of convection/advection, the reaction–diffusion equations are given by
2.1
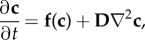
where 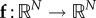 is in general a nonlinear system for the rate equations of a CRN involving *N* species (*X*_1_, …, *X*_*N*_), and **D** is a diagonal matrix containing the diffusion rates of each species. The ∇ operator describes the spatial derivatives in 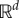, where *d* is the number of spatial dimensions. In one dimension, this simply corresponds to ∂^2^**c**/∂*x*^2^.

A Turing pattern arises when an equilibrium of the spatially homogeneous system (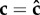 such that 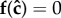) goes unstable in the presence of diffusion. In our definition of a Turing pattern, this equilibrium is also assumed to be stable in the absence of diffusion. To analyse stability, we consider standard linear analysis of the system about the equilibrium 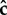. If 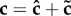 when 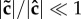, then (2.1) becomes
2.2
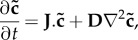
where **J** is the matrix of the first-order partial derivatives of **f** with respect to each species *j*
2.3
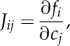
evaluated at 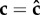.

To assess stability in the presence of diffusion, we consider how perturbations evolve over time. If *w*_*k*_(**x**) are the eigenmodes of the Laplacian operator ∇^2^, i.e. ∇^2^*w*_*k*_ = *η*_*k*_*w*_*k*_, then it has been shown that *η*_*k*_ ≤ 0 (with zero flux boundary conditions) [30]. Therefore, it is customary to let *η*_*k*_ =− *k*^2^, with *k* corresponding to the wavenumber of the eigenmode. As such, in one dimension, on a domain *x* ∈ [0, *L*], there are solutions of the form
2.4
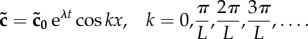
Accordingly, the original linearization problem (2.2) translates into
2.5
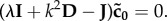
Therefore, we are interested in the eigenvalues of **J** − *k*^2^**D**. If we denote by *σ*_**J**−*k*^2^__**D**_ the spectrum of **J** − *k*^2^**D**, then this gives rise to a dispersion relation
2.6
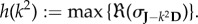


For Turing instability, we require that the system is stable in the absence of diffusion, which translates to eigenvalues at *k* = 0 all having negative real part. Additionally, we require the existence of at least one unstable wavenumber, i.e. there exists a wavenumber k* such that there is a corresponding eigenvalue λ* with positive real part.

### 2.2. Model reduction

We now consider a system of *N* species in which the first *M* species can diffuse with diffusion coefficients *D*_1_, …, *D*_*M*_, while the remaining species cannot. Accordingly, we describe a spatially inhomogeneous reaction–diffusion system as
2.7
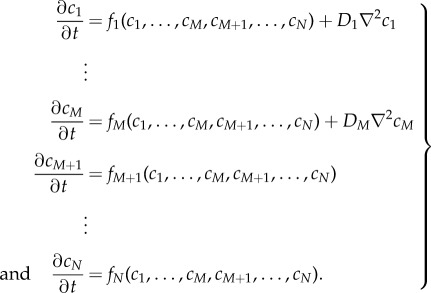
The associated spatially homogeneous system can therefore be written compactly as
2.8
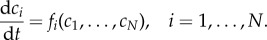
Because we look for Turing patterns we further assume that there exists a non-negative spatially homogeneous equilibrium of (2.1) given by 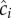, which satisfies
2.9
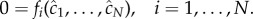


We now outline a strategy to reduce system (2.8) to a smaller system of *N*_*R*_ species, with *M* ≤ *N*_*R*_ < *N*, including all diffusible species. Without loss of generality, we assume that the reduced model consists of species *X*_1_,…, *X*_*N*_*R*__. The reduction is obtained by defining the functions 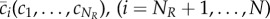, which satisfy
2.10
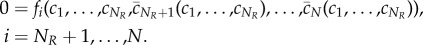
Intuitively, this amounts to solving the steady-state ODEs for the *removed* species, as functions of the *remaining* species, thereby eliminating *N* − *N*_*R*_ species from the system. Note that 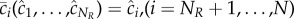. The reduced system of ODEs becomes
2.11
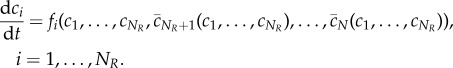
Using the chain rule, the Jacobian of the reduced system (2.11) is the *N*_*R*_ × *N*_*R*_ diagonal matrix given by
2.12
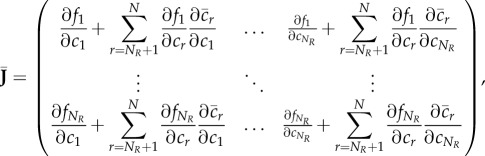
evaluated at 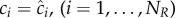. Accordingly, the diffusion matrix of the reduced system (2.11) is the *N*_*R*_ × *N*_*R*_ diagonal matrix given by
2.13
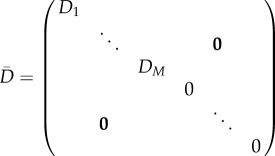
where the first *M* diagonal entries are the diffusion rates of the diffusible species, and the additional *N*_*R*_ − *M* entries correspond to non-diffusible species that were not removed during the model reduction.

We now have two systems, (2.8) and (2.11), which are models of the same underlying process. To consider how Turing pattern formation is affected by the reduction from (2.8) to (2.11), we return to the mathematical conditions of pattern-forming behaviour introduced above.

We say a system is *pattern-forming* if there exist *k*_1_, *k*_2_, *k*_3_, *k*_4_ > 0 with *k*_1_ ≤ *k*_2_ < *k*_3_ ≤ *k*_4_ such that all eigenvalues of **J** − *k*^2^**D** have negative real parts when *k* < *k*_1_ and *k* > *k*_4_, and there is a positive real eigenvalue when *k*_2_ < *k* < *k*_3_. This is a strict definition that explicitly excludes certain systems that are capable of forming patterns: (i) systems with patterns formed by Turing–Hopf bifurcations, (ii) systems that are unstable without diffusion, and (iii) systems that can form patterns on arbitrarily small length-scales (‘noise-amplifying networks’ [11]). Systems of type (i) are excluded because they can form either spatial patterns or temporal oscillations depending on the initial conditions, and so are not consistently pattern-forming; systems of type (ii) are excluded because they violate the concept of DDI; systems of type (iii) are excluded because they violate physical principles by permitting, for example, patterns on length-scales smaller than a molecule [10].

Knowing that we are interested in the behaviour of the matrix **J** − *k*^2^**D**, and its reduced counterpart 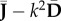, we note the following relationship between the full and reduced systems.

**Lemma 2.1.**

|**J** − *k*^2^**D**| *and* 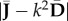 *change sign at the same values of* *k*.

**Proof.**

We define
2.14
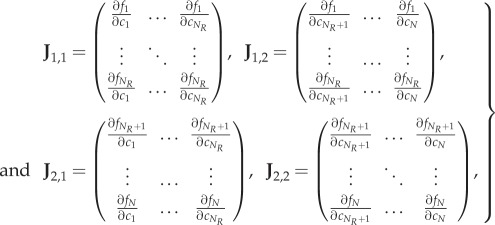
so that
2.15
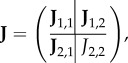
and we define
2.16
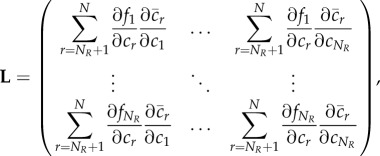
so that
2.17
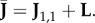
We now compare |**J** − *k*^2^**D**| and 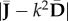. In the former case, the Schur complement of **J**_2,2_ provides the relationship
2.18
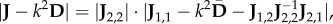
while in the latter case
2.19
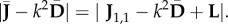
The two determinants are directly proportional if **L** = −**J**_1,2_ **J**^−1^_2,2_ **J**_2,1_. We observe that we can write 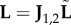, where
2.20
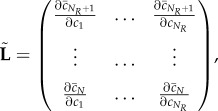
so that the condition for proportional determinants becomes that 
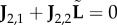
, or algebraically, that
2.21
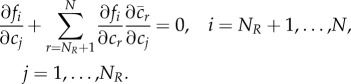
We note from equation (2.10) that, 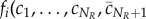 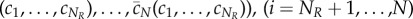 is a constant function of *c*_1_, …, *c*_*N*_*R*__, i.e.
2.22
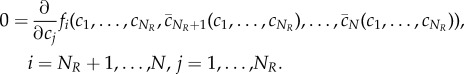
Expanding this gives precisely the condition (2.21). It follows that the determinants of the full and reduced systems are directly proportional, and consequently change sign at exactly the same values of *k*. □

We next turn our attention to the conditions of Turing pattern formation, specifically considering when eigenvalues can cross the imaginary axis. We can make the following statement.

**Lemma 2.2.**

*A system is pattern-forming if it has the following properties*:

(*I*) *the system is linearly stable without diffusion* (*i.e.* 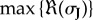),
(*II*) *the non-diffusible subsystem is either linearly stable without diffusion* (i.e. 
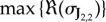
), *or else non-existent* (*i.e.* *M* = *N*),
(*III*) |**J** − *k*^2^**D**| *changes sign at least twice as a function of* *k*, *and* *we denote smallest two such values of* *k* *as* *k*_2_ *and* *k*_3_, *with* 0 < *k*_2_ < *k*_3_.

**Proof.**

By (I), the real parts of eigenvalues of **J** − *k*^2^**D** are negative when *k* = 0, and by continuity, also negative up to some *k*_1_ > 0 with *k*_1_ ≤ *k*_2_. When *k* is very large, the characteristic polynomial of the system will have the form (*λ* + *k*^2^*D*_1_)(*λ* + *k*^2^*D*_2_) ⋯ *s*(*λ* + *k*^2^*D*_*M*_)|*λ***I** − **J**_2,2_| + *O*(*k*^2*M*−2^) = 0 if *N* > *M*, or else (*λ* + *k*^2^*D*_1_)(*λ* + *k*^2^*D*_2_) ⋯ *s*(*λ* + *k*^2^*D*_*M*_) = 0 if *N* = *M*. The eigenvalues of **J** − *k*^2^**D** will therefore converge to −*k*^2^*D*_1_, −*k*^2^*D*_2_, …, −*k*^2^*D*_*M*_ and if *N* > *M*, also the eigenvalues of **J**_2,2_, which all have negative real part, by (II). It follows that all eigenvalues of **J** − *k*^2^**D** have negative real part for sufficiently large *k* (say, larger than some *k*_4_ ≥ *k*_3_). Furthermore, by (III), |**J** − *k*^2^**D**| changes sign first at *k*_2_ and second at *k*_3_. As there exist *k* < *k*_2_ and *k* > *k*_3_ both corresponding to all negative real part eigenvalues of **J** − *k*^2^**D**, it follows that there is at least one eigenvalue with positive real part when *k*_2_ < *k* < *k*_3_. The system therefore satisfies all conditions required for pattern-forming behaviour. ▪

The combination of lemmas 2.1 and 2.2 directly provides the conditions for which model reduction preserves pattern-forming behaviour. In particular, we have the following result.

**Lemma 2.3.**

*If a full* (*reduced*) *system is pattern-forming, then the reduced* (*full*) *system is also pattern-forming if both the reduced* (*full*) *system and—if it exists—its non-diffusible subsystem are stable without diffusion.*

**Proof.**

Conditions (I) and (II) of lemma 2.2 hold by definition. As the full (reduced) system is pattern-forming, there must exist distinct smallest values of *k*, *k*_2_ and *k*_3_, with 0 < *k*_2_ < *k*_3_ such that |**J** − *k*^2^**D**| changes sign at them. By lemma 2.1, the full and reduced systems change signs at the same values of *k*, so condition (III) of lemma 2.2 also holds. Therefore, the reduced (full) system is pattern-forming. ▪

There are two important implications of this result for model reduction in practice. Firstly, if we reduce a large model and find a set of parameters for which the reduced model forms patterns, then we only have to check the Jacobian of the full model to find if it also forms patterns. This is useful because checking the stability of a Jacobian is computationally much simpler than finding largest real eigenvalues as functions of *k*, especially for systems with many species. Secondly, if the reduced model is stable for a region of parameter space, then the full model cannot form patterns in that region. This is useful because the stable region is typically large, and unstable regions frequently correspond to physically impossible parameter values, and so model reduction can be an efficient way of eliminating systems incapable of pattern formation.

In the next section, we apply our technique to some example systems and confirm that our results hold.

## 3. Examples

### 3.1. Brusselator

One of the simplest chemical systems that is known to exhibit Turing patterns is the Brusselator, which in its original form is described by four reactions involving only two essential chemical species *X* and *Y* [31]
3.1
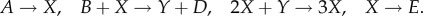
Here, the species *A*, *B*, *D* and *E* are explicitly included to ensure that mass is conserved. However, they are often removed during analysis as they do not contribute to the characterization of the system behaviour, under the assumption that *A* and *B* are never depleted.

Following some debate over the chemical plausibility of reactions with more than two reactants, it was proposed in [32] that by introducing a third chemical species, the trimolecular reaction could be converted to a pair of bimolecular reactions
3.2
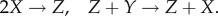


As the resulting bimolecular Brusselator system has not previously been analysed for Turing pattern formation explicitly, we applied our model reduction approach to determine conditions for which Turing instability is preserved. To simplify the reaction network while retaining full coverage of the space of possible behaviours of the bimolecular Brusselator system, we remove the non-essential species and remove two of the rate parameters, leaving
3.3
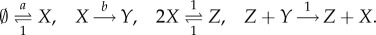
Note that we allow for the first new reactions to be reversible (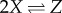), but keep the subsequent reaction irreversible, which together produces an essentially irreversible transition from 2*X* + *Y* to 3*X*, as in the original scheme. Assuming that *X* diffuses with unit rate, *Y* at a relative rate *D*_*Y*_ and *Z* is immobile, the concentrations of *X*, *Y* and *Z* for this system evolve as
3.4
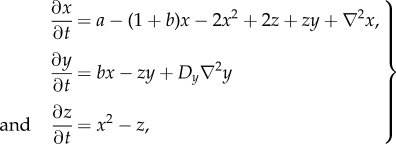
with equilibria 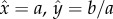, 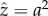. We perform a model reduction which removes *Z* from the system. As per our strategy, this is achieved by solving d*z*/d*t* = 0 for 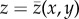. We get
3.5
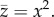
The reduced model is obtained by substituting equation (3.5) into equation (3.4)
3.6
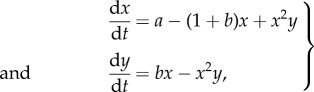
which recovers the reaction–diffusion equations for the classical Brusselator model.

**Figure 1.**
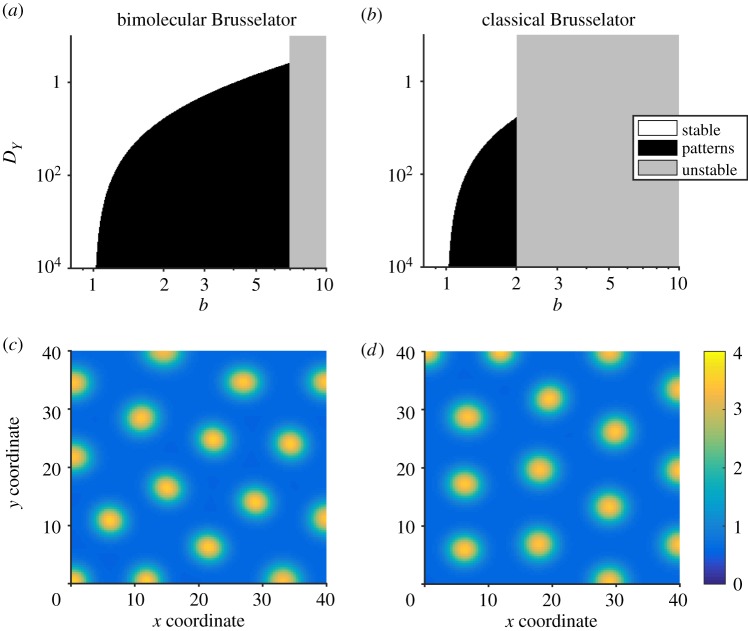
Model reduction of a bimolecular Brusselator mostly retains the bifurcation structure of the classical Brusselator model. Bifurcation diagrams are shown for (*a*) the bimolecular Brusselator (3.4) system and (*b*) the classical Brusselator (3.6) system. The clear area indicates parameter values of *b* and *D*_*Y*_ for which the homogeneous equilibrium is stable, while the black region indicates parameter values corresponding to Turing instability. The grey region indicates where the equilibrium solution is unstable in the spatially homogeneous scenario. (*c*,*d*) Stable patterns formed by species *X* in the bimolecular Brusselator (3.4) and classical Brusselator (3.6) systems respectively. In the bimolecular Brusselator, *Z* is assumed to be non-diffusible, which enables its removal by our model reduction procedure. The parameter values used in these analyses were *a* = 1, *D*_*X*_ = 1, *b* = 1.88, *D*_*Y*_ = 10. Spatial simulations used a domain length of 20 (arbitrary units).

In general, we find that parameter values which lead to patterns in the bimolecular Brusselator model also lead to patterns in the classical Brusselator model (figure 1*a*,*b*). To see this, we varied the parameter *b* and the diffusion constant *D*_*Y*_ over large ranges, and compared the bifurcation diagrams. As we would expect from lemma 2.3, these show that parameter values which lead to patterns in the reduced system also lead to patterns in the full system; correspondingly, parameters which lead to patterns in the full system lead either to patterns or instability in the reduced system. In figure 1*c*,*d*, we show the patterns formed by the species *X* in systems (3.4) and (3.6), respectively.

**Figure 2.**
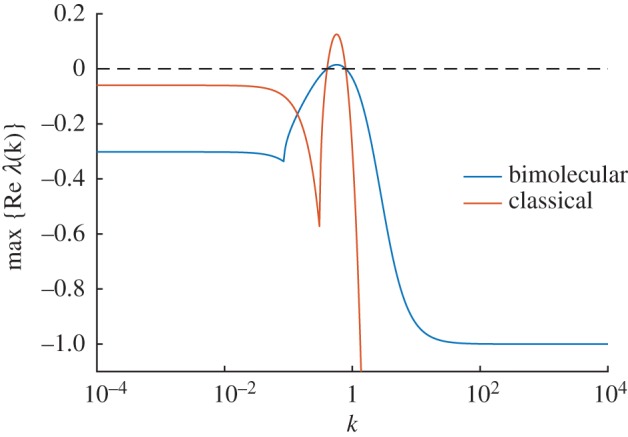
Dispersion relations for the bimolecular and classical Brusselators. Dispersion relations are shown for the bimolecular Brusselator (3.4) system and the classical Brusselator (3.6) system. Although the two plots are different in general, they both cross the zero line at the same points. Parameter values are as in figure 1.

In figure 2, we show the dispersion relations for systems (3.4) and (3.6). As predicted by lemma 2.1, while the relations themselves are different, they both change sign at the same values of *k*, implying that both systems will form patterns on the same wavelengths.

### 3.2. Turing's example

We next considered a larger example that is closely related to one proposed by Turing [8]. It consists of species *X*,  *Y*,  *W*,  *C* and *C*′, and concentrations *x*,  *y*,  *w*,  *c* and *c*′ respectively. *X* and *Y* can diffuse with diffusion coefficients *D*_*X*_ and *D*_*Y*_, and the reactions are given by
3.7
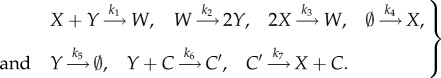
We note that the *C* and *C*′ are related via a conservation law, and so we substitute *c*′ = *c*_Tot_ − *c* (*c*_Tot_ constant), which leads to four independent ODEs that completely characterize the deterministic behaviour of the system
3.8
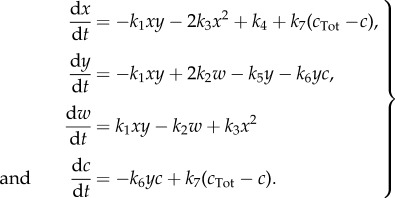


To demonstrate how the model reduction can be applied to different extents, we reduce this system to both 3- and 2-species system approximations. First, we eliminate *c* by solving d*c*/d*t* = 0 for 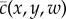, which gives
3.9
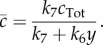
Substituting 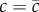 in system (3.8), we obtain a three species system defined by
3.10
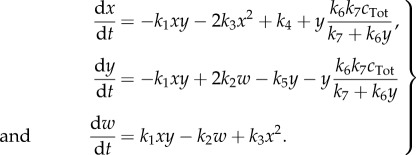
Next, we eliminate *w* by solving d*w*/d*t* = 0 for 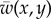, which gives
3.11
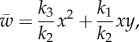
Substituting 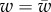 in (3.10), we obtain a two species system defined by
3.12
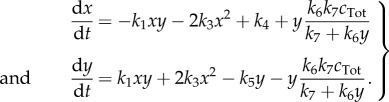


**Figure 3.**
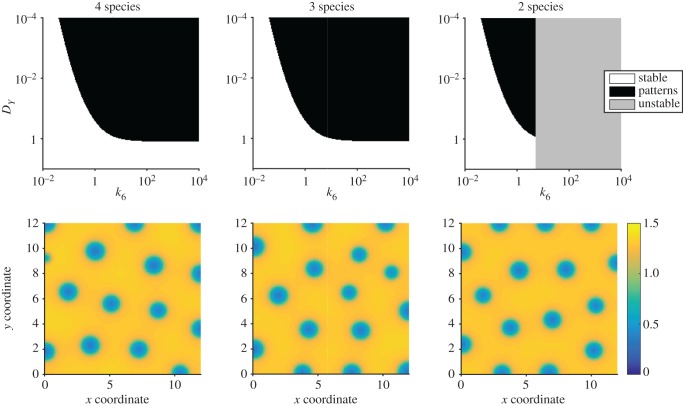
Turing pattern analysis for a reaction network from Turing [8]. (*a*–*c*) Bifurcation diagrams for 4- (3.8), 3- (3.10) and 2-species (3.12) systems. In all cases, the parameter values used were *k*_1_ = 2, *k*_2_ = 0.2, *k*_3_ = 0.01, *k*_4_ = 0.08, *k*_5_ = 0.04, *k*_7_ = 2, *c*_Tot_ = 6, *D*_*X*_ = 1. (*d*–*f*) Stable patterns formed by species *X* in the 4- (3.8), 3- (3.10) and 2-species (3.12) systems, respectively. In all cases, the parameter values used were as in (*a*–*c*) but additionally *k*_6_ = 3.37, *D*_*Y*_=0.04, domain length = 6.

We therefore arrive at three models of system (3.7) with varying levels of dynamical complexity. A *complete* model is described by four species, whereas two successive equilibrium assumptions applied to *C* and then *W* generate two simpler models. To demonstrate the equivalence of Turing instability (lemma 2.3) across these models, we illustrate bifurcation diagrams of the full system (figure 3*a*), and the reduced three (figure 3*b*) and two species (figure 3*c*) systems. These show that the four and three species models have indistinguishable parameter-dependent behaviour, while the two species model is unstable for a region of parameter space where the other models are stable. We note that this unstable region prevents the two species model from forming patterns when the diffusion rates of *X* and *Y* are equal (*D*_*X*_ = *D*_*Y*_ = 1), though such equal diffusion rates can produce patterns for the three and four species models. We also observe that pattern-forming parameters in the two species system also lead to patterns in the larger systems (figure 3*d*–*f*). This is similar to the situation observed for the Brusselator, whereby model reduction leads to a shrinkage of the parameter space that produces patterns. Intuitively, adding immobile species should lead to an expansion of the permissible parameter space in general, and therefore we are observing the opposite of this when applying our form of model reduction.

**Figure 4.**
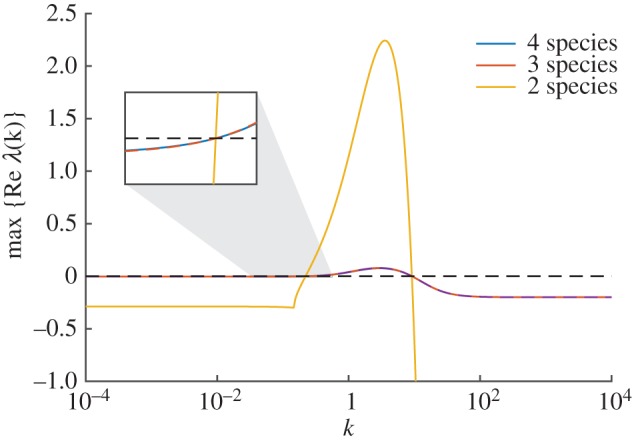
Dispersion relations for the reaction network from Turing [8]. Dispersion relations are shown 4- (3.8), 3- (3.10) and 2-species (3.12) systems (blue, red and yellow, respectively). Although the plots are different in general, all three cross the zero line at the same points. An inset plot shows the crossing of the zero line at the lower value of *k*. Parameter values are as in figure 3.

In figure 4, we show the dispersion relations for the 4- (3.8), 3- (3.10) and 2-species (3.12) systems. As predicted by lemma 2.1, while the relations themselves are generally different (although the 3- and 4-species relations are near-indistinguishable), they all change sign at the same values of *k*, implying that all systems will produce patterns on the same length scales.

### 3.3. A synthetic gene circuit

In our final example, we consider a much larger system consisting of 17 species, which is based on the synthetic gene circuit proposed in [18]. While this publication presents only a theoretical analysis of the synthetic gene circuit, it represents a biologically plausible approach to realizing a synthetic cellular Turing patterning circuit in live cells. The synthetic gene circuit is arranged in an activator–inhibitor network, whereby the intercellular signalling molecule acyl homoserine lactone (AHL) plays the role of a short-range activator, and hydrogen peroxide gas (H_2_O_2_) plays the role of a long-range inhibitor. Activation is achieved by AHL binding a constitutively expressed LuxR receiver protein, forming an activating complex for PLux promoters, which are placed upstream of coding sequences for the AHL synthase luxI [33] and the H_2_O_2_-producing ndh. The inhibitory loop is formed by an H_2_O_2_-sensitive topA promoter stimulating production of the AHL lactonase aiiA, which degrades AHL [34], thus inhibiting its action.

In [18], it is shown that a model containing five variables (but analysis over four variables due to the presence of a conservation law) can give rise to DDI for certain parameter choices. Already, analysis of Turing instability is made challenging by virtue of there being more than two essential dependent variables. One might categorize their model as having intermediate complexity, as a simpler model could be arrived at by considering only the concentrations of the diffusive signals AHL and H_2_O_2_. By contrast, a more complex model might be considered that describes more of the intracellular components, and complexes between them, directly.

Here, we show that the bifurcation properties of models of the synthetic gene circuit in [18] are preserved across models of varying complexity. To demonstrate this, we start by considering a model described by elementary chemical reactions, as follows:


3.13
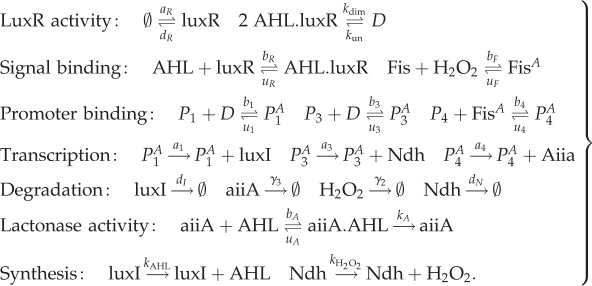


For brevity, we do not write out the full system of reaction rate equations here (although code is available from the authors upon request). We reduce the full system of equations to one of intermediate complexity consisting only of AHL, H_2_O_2_, AHL.luxR and aiiA (as considered in [18]), whose concentrations we write as *L*, *H*, *P* and *A*, respectively. The reduced ODEs are


3.14
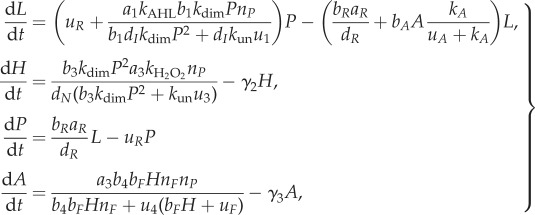


where *n*_P_ is the total promoter concentration and *n*_F_ is the total *Fis* concentration. While this system is very similar to the four species model studied in [18], there are some minor differences, but we nevertheless still find that Turing instabilities arise. These differences arise because we arrived at equation (3.14) by reducing a full mass-action system equation (3.13), while the system in [18] is not obtained systematically from a more complex model.

Finally, we can further reduce the system of intermediate complexity to a system comprising only the diffusive molecules AHL and H_2_O_2_:


3.15
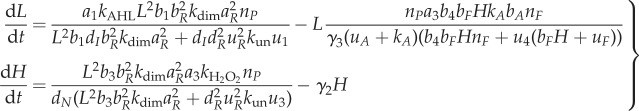


**Figure 5.**
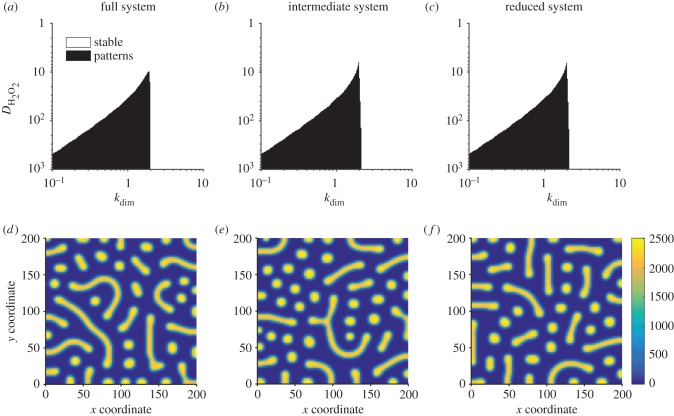
Turing patterns are robust to reductions of a model of a synthetic gene circuit with intercellular signalling. (*a*–*c*) Bifurcation diagrams for systems (3.13)–(3.15), respectively. (*d*–*f*) Stable patterns formed by species AHL in systems (3.13)–(3.15), respectively. Parameter values: *a*_1_ = 2142, *a*_3_ = 1190, *k*_AHL_ = 2, *k*_H_2___O_2__ = 0.057,  *b*_1_ = 0.156,  *b*_3_ = 0.03, *b*_4_ = 0.25,  *b*_*F*_ = 2,  *d*_*N*_ = 2,  *d*_*I*_ = 2, *d*_*R*_ = 2,  *γ*_2_ = 2,  *γ*_3_ = 2,  *b*_*R*_ = 0.0156, *u*_*R*_ = 2,  *u*_*A*_ = 2,  *k*_*A*_ = 2,  *b*_*A*_ = 0.0117, *a*_*R*_ = 0.5,  *k*_un_ = 2,  *n*_*F*_ = 2,  *n*_*P*_ = 2,  *u*_1_ = 2,  *u*_3_ = 2,  *u*_4_ = 2, *u*_*F*_ = 2,  *D*_AHL_ = 1; in (*d*–*f*), *k*_dim_ = 2, *D*_H2O2_ = 100, domain length = 100.

**Figure 6.**
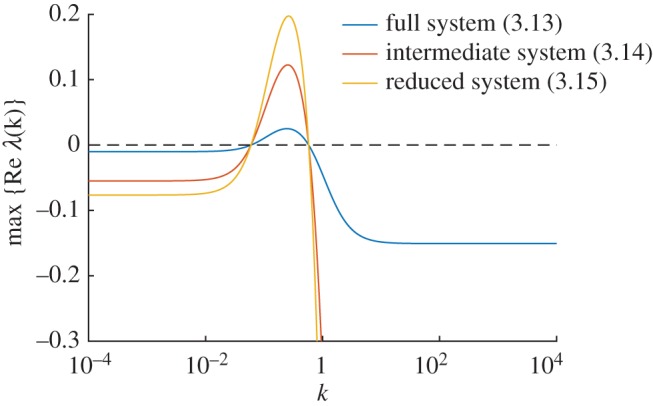
Dispersion relations for the synthetic gene circuit model. Dispersion relations are shown for systems (3.13)–(3.15) (blue, red and yellow, respectively). Although the plots are different in general, all three cross the zero line at the same points. Parameter values are as in figure 5.

In figure 5*a*–*c*, we show bifurcation diagrams of the full system (3.13), the four species model (3.14) and the two species model (3.15). In this case, we find that the diagrams for the full (3.13) and intermediate (3.14) complexity systems are identical, while the diagram for fully reduced system (3.15) shows an unstable region of parameter space where the larger models are stable (in accordance with lemma 2.3). All three models have identical pattern forming regions. In figure 5*d*–*f*, we show stable two-dimensional patterns of [AHL] in each system, which illustrates how patterns of a similar wavelength emerge. This is confirmed by the dispersion relations shown in figure 6, which show that each system's dispersion relation changes sign at precisely the same wavenumbers (in accordance with lemma 2.1).

## 4. Discussion

In this paper, we have proposed a technique for reducing a large chemical system to a small one, in a manner that preserves pattern-forming behaviour as far as possible. In essence, the reduction relies on a QSSA, since it assumes that the concentrations of the removed species can be written in terms of the remaining species without reference to time. However, the QSSA is a method to eliminate species which can be considered to be in equilibrium, to simplify the description of the non-equilibrium species; in Turing patterning systems all species are necessarily in equilibrium, so it may be misleading to equate our reduction method with the QSSA, although they are mathematically equivalent. Comparison with other model reduction techniques is also difficult, as unlike typical approaches, our strategy is not necessarily interested in preserving the correct dynamical behaviour of the full system: indeed, we make no claims that the reduced model is an accurate description of the original system. But as a consequence, it is notable that our approach does not require specific values of kinetic parameters, unlike model reductions in other systems and their behaviours.

The reduced model is derived with one aim in mind: to help find parameters of the full model which can lead to Turing patterns, or to help prove that none exist. In that respect, our technique is very successful. We have shown that, if we can find a pattern-forming parameter set for the reduced system, then it is simply a matter of checking the stability of the Jacobian of the full system to determine whether it, too, forms patterns with those parameters. Furthermore, if we can find a region of parameter space for which the reduced system is stable, then we know for certain that the full system cannot form patterns in that region.

The power of our technique is demonstrated very well on system (3.13): a biologically plausible system consisting of 17 species and 31 reactions. At face-value, it is impossible to know whether this system is capable of forming patterns, and, if so, which parameters correspond to pattern-forming behaviour. By performing a dramatic reduction from 17 to 2 species, due to there only being two diffusive species, we quickly found regions of parameter space corresponding to pattern-forming and stability in the reduced model. Our results prove that these regions necessarily correspond to potential-pattern-forming and no-pattern-forming, respectively, in the full system. The fact that both systems generate near-indistinguishable patterns is an added bonus. Temporal dynamics are not conserved, which can be seen in the larger eigenvalues of reduced systems (figures 2, 4 and 6), which is known to correlate with faster pattern emergence [35]. However, this is not surprising. Our model reduction technique is to simply assume that certain species equilibrate infinitely fast, and so the overall dynamics of reduced systems will be faster, in general.

Overall, our results provide a quick and rigorous way to check for pattern-forming behaviour in large biochemical networks. While we do not attempt to automate this process here, such automation could be of serious utility to synthetic biologists in their attempts to find and synthesize genetic networks capable of forming stable patterns. While the results here are unfortunately not applicable to the purely diffusive systems, we feel they will be of general interest to those in the reaction–diffusion field, as they provide a means to extend the current analytical tools developed for two or three species Turing-patterning systems to systems that additionally include an arbitrary number of non-diffusible species.

## 5. Numerical methods

All simulations and figures were prepared using Matlab R2016a. Code is available from GitHub: https://github.com/ndalchau/turing-model-reduction.

### 5.1. Dispersion relations

Dispersion relations (figures 2, 4 and 6) were evaluated numerically using Matlab's built-in eigenvalue function eig. The eigenvalues were computed for *J* and *J* − *k*^2^*D* (as defined in §2.1), with the equilibrium points as specified in the text of each example, and Jacobian entries differentiated by hand.

### 5.2. Bifurcation diagrams

Bifurcation diagrams (figures 1*a*,*b*, 3*a*–*c* and 5*a*–*c*) were constructed by sampling parameter values uniformly in two-dimensional subspaces of the overall parameter space for each example. Then for each parameter set, a dispersion relation was numerically evaluated. Region colours were then assigned according to the sign of the maximum of the real part of the eigenvalues at each wavenumber.

### 5.3. PDE simulations

PDE simulations (figures 1*c*,*d*, 3*d*–*f* and 5*d*–*f*) were carried out using an explicit finite difference scheme on a regular grid. The code was implemented in Matlab R2016a, making use of the del2u function to generate a finite difference approximation of ∇^2^ at each time step.

## Data accessibility

Code for reproducing the numerical results is available from https://github.com/ndalchau/turing-model-reduction.
